# Protein Painting Mass Spectrometry in the Discovery of Interaction Sites within the Acetylcholine Binding Protein

**DOI:** 10.1021/acschemneuro.4c00149

**Published:** 2024-05-28

**Authors:** Alexandru Graur, Amanda Haymond, Kyung Hyeon Lee, Franco Viscarra, Paul Russo, Alessandra Luchini, Mikell Paige, Isabel Bermudez-Diaz, Nadine Kabbani

**Affiliations:** † School of Systems Biology, George Mason University, Fairfax, Virginia 22030, United States; ‡ Center for Applied Proteomics and Molecular Medicine, George Mason University, Manassas, Virginia 20110, United States; § Department of Chemistry and Biochemistry, George Mason University, Fairfax, Virginia 20110, United States; ∥ Department of Biological and Medical Sciences, Faculty of Health and Life Sciences, Oxford Brookes University, Headington, Oxford OX3 0BP, United Kingdom; ⊥ Structural Bioinformatics and Computational Biochemistry, Department of Biochemistry, University of Oxford, Oxford OX1 3QU, United Kingdom

**Keywords:** nicotinic receptor, ligand binding, bungarotoxin, amyloid, protein interactions

## Abstract

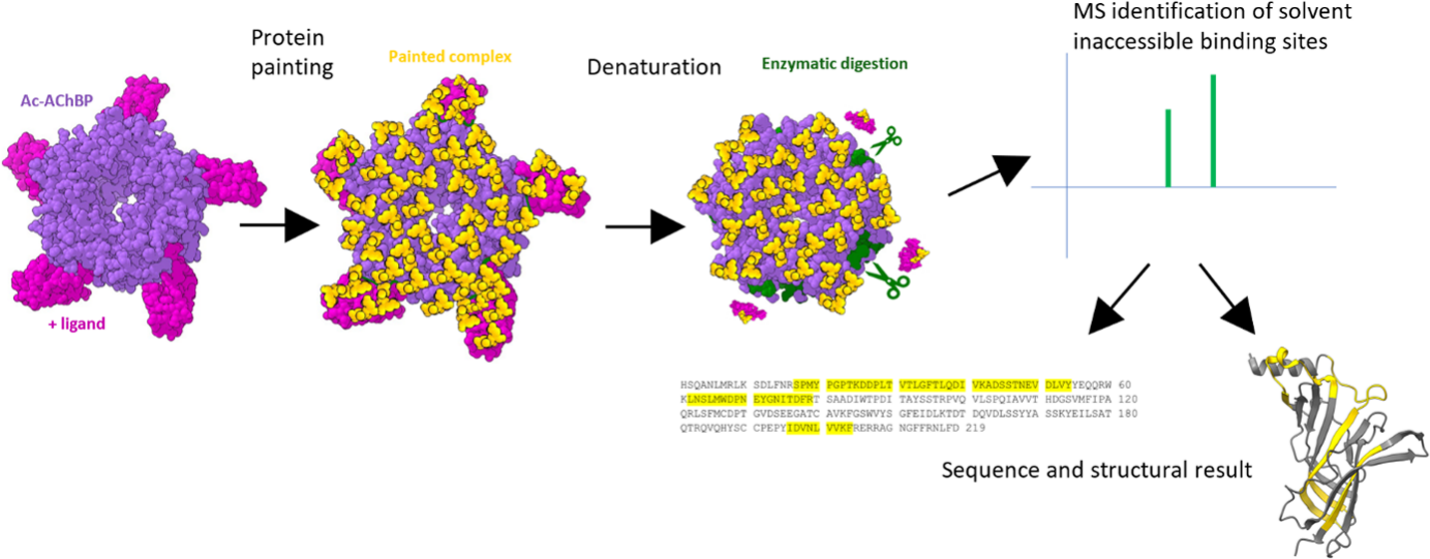

Nicotinic acetylcholine receptors (nAChRs) are a family of ligand-gated ion channel receptors that contribute to cognition, memory, and motor control in many organisms. The pharmacological targeting of these receptors, using small molecules or peptides, presents an important strategy for the development of drugs that can treat important human diseases, including neurodegenerative disorders. The *Aplysia californica* acetylcholine binding protein (Ac-AChBP) is a structural surrogate of the nAChR with high homology to the extracellular ligand binding domain of homopentameric nAChRs. In this study, we optimized protein-painting-based mass spectrometry to identify regions of interaction between the Ac-AChBP and several nAChR ligands. Using molecular dyes that adhere to the surface of a solubilized Ac-AChBP complex, we identified amino acid residues that constitute a contact site within the Ac-AChBP for α-bungarotoxin, choline, nicotine, and amyloid-β 1–42. By integrating innovation in protein painting mass spectrometry with computational structural modeling, we present a new experimental tool for analyzing protein interactions of the nAChR.

## Introduction

Mammalian nicotinic acetylcholine receptors (nAChRs) are composed of five subunits, with neuronal subunits categorized into two main groups: α (α2 to α7, α9, and α10) and β (β2−β4). They can be distinguished by the presence of adjacent cysteine groups exclusively in the extracellular domain of the α subunits.^1^ Similar to other ligand-gated ion channels, nAChRs undergo a transition between several structural states: nonligand bound closed, ligand bound open, and ligand bound nonconducting desensitized state.^2,3^ The complexity of nAChR signaling is further augmented by the potential for a large number of combinatorial subunit arrangements within the pentameric channel.^4^ Homopentameric nAChRs such as α7 and α9 types are unique in both stoichiometry and high calcium conductance properties.^5−7^ Of these homopentameric nAChRs, α7 is a promising molecular target for the development of new drugs for various human diseases including neurodegeneration and cancer.^8^

α7 nAChRs are activated by various types of ligands including natural small molecules such as choline and nicotine, as well as synthetic compounds such as PNU-282987.^9−11^ α7 nAChRs are also activated by peptides of varying sequence lengths and structural conformations. For example, proteins exhibiting a three-finger toxin (3ftx) receptor-targeting fold are shown to bind tightly to nAChRs including α7 homopentamers.^12,13^ Such proteins include protoxins, such as lynx1, and neurotoxins, such as α-conotoxin ImI and α-bungarotoxin (Bgtx).^14,15^ A 3ftx-fold has also been suggested within some human disease-causing proteins including the SARS-CoV2 spike glycoprotein.^16^

Knowledge of the structural underpinnings of ligand binding sites within nAChRs has been significantly propelled by studies involving the *Aplysia californica* acetylcholine binding protein (Ac-AChBP). Ac-AChBP is a water-soluble pentameric protein complex that structurally resembles the extracellular domain of homopentameric nAChRs and maintains an ability to bind many nAChR ligands including small molecules and peptides.^17^ Ac-AChBP is a valuable structural surrogate in crystallography, NMR, and cryo-electron microscopy (cryo-EM) studies of the nAChR.^18−22^ Ac-AChBP shares important configurational homologies with the nAChR including various loops and β-strands enriched in aromatic residues (e.g., Tyr and Trp) that contribute to a ligand binding aromatic nest.^23,24^ In this study, we use a recently developed protein painting mass spectrometry (MS) technique to identify ligand interaction sites within the Ac-AChBP. We assessed the ability to detect binding to several canonical ligands for homopentameric nAChRs, including choline and Bgtx, and explored the ability to identify sites for association with amyloid β 1−42 (Aβ42) that has been shown to bind α7 nAChRs.^25^ Protein painting is presented as a new strategy in the study of protein–ligand interactions for the nAChR.

## Results

### Expression and Optimization of Protein Painting to Study Interactions of Ac-AChBP

**Figure 1:**
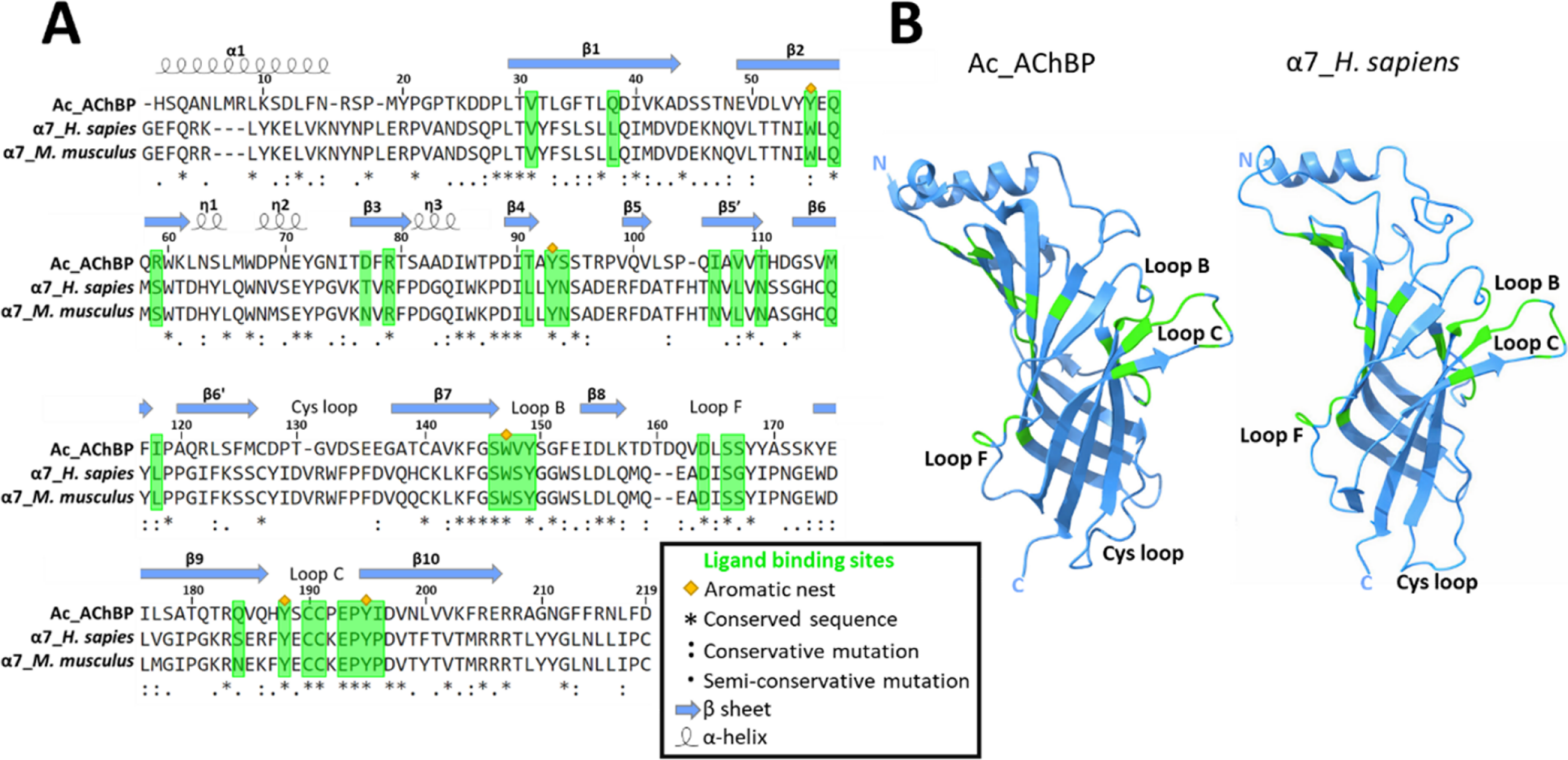
Sequence and structural similarity of Ac-AChBP and α7 nAChRs. (A) Multisequence alignment showing the location of binding motifs within Ac-AChBP (UniProt; Q8WSF8; aa position 1–219), human α7 nAChR (UniProt; P36544; aa positions 23–241), and mouse α7 nAChR (UniProt; P49582; aa positions 23–241). (B) Structural model of the Ac-AChBP and human α7 nAChR.

Ac-AChBP serves as a valuable tool for investigating the ligand binding properties of nAChRs based on its strong sequence and structural homology at the extracellular ligand binding site including multiple loops and residues that make up the aromatic nest within the AChBP and nAChRs^22−24^ (Figure 1). Various structures of the AChBPs, in complex with ligands, are available in PDB.^26^

**Figure 2:**
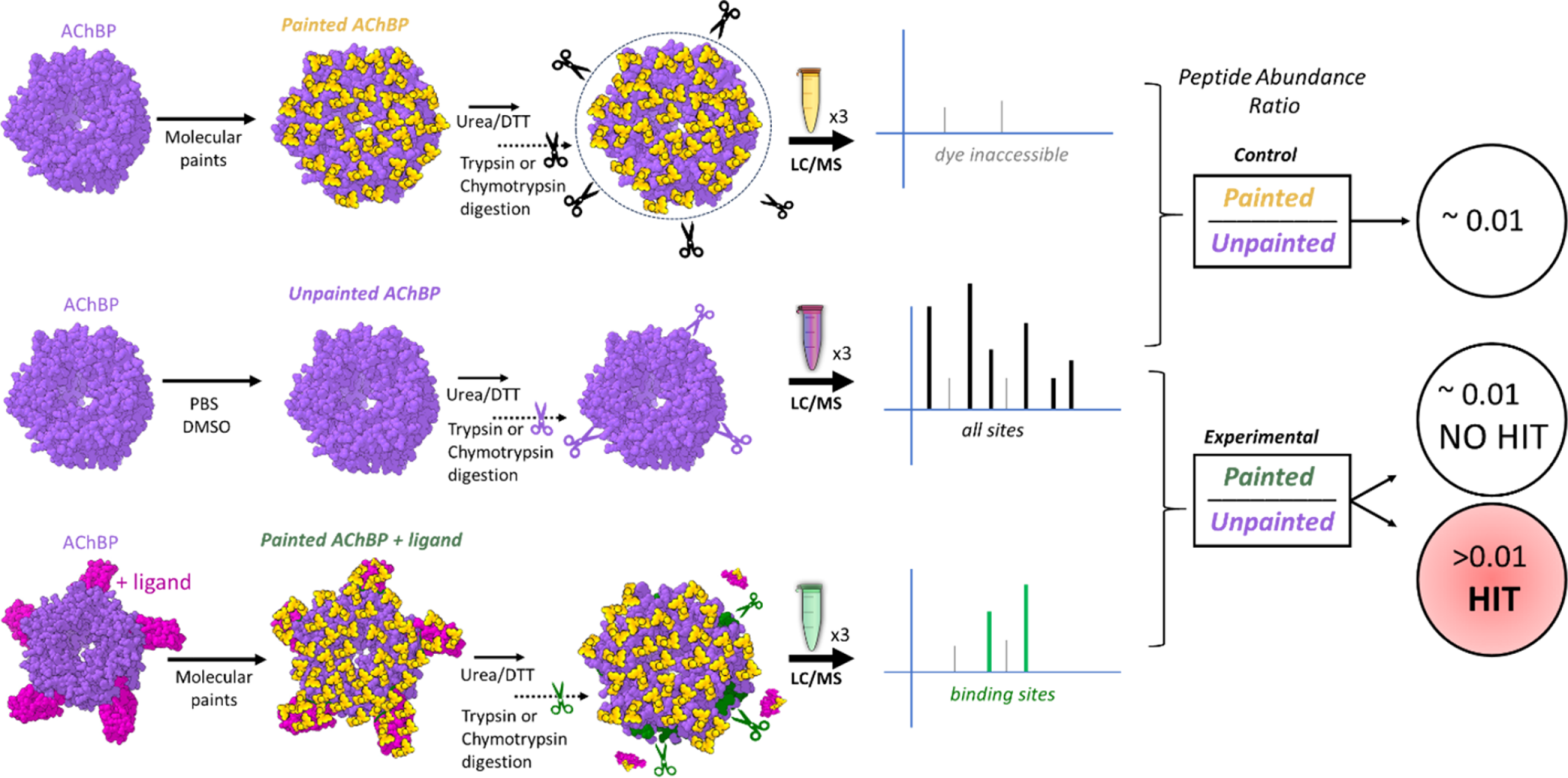
Summary of the experimental design. A protein painting method was adapted for the study of ligand binding within the Ac-AChBP.^28,29^

We used Ac-AChBP in a protein painting MS for the study of ligand interactions within soluable macromolecular complexes.^27−29^ An overview of the protein painting study is shown in Figure 2. We developed the protein painting strategy through identifying optimal conditions of dye application, peptidase digestion, and detection of enzymatic digestion fragments (EDF). The protein paint assay enables MS identification of EDF across painted and nonpainted samples as well as between ligand and no ligand conditions. Positive EDF hits in the painted Ac-AChBP experiment are determined through a statistical measure of significance between their abundance ratio in the ligand present to ligand absent experimental conditions.

**Figure 3:**
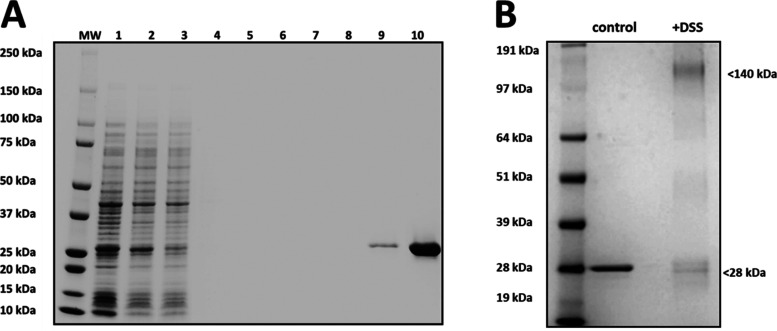
Detection of the Ac-AChBP. (A) Coomassie-stained SDS-PAGE gel showing induction and purification of 6xHis-tagged Ac-AChBP from BL21 cells. Lanes: 1, crude lysate; 2, protein fraction, 3, Ni-NTA column bound fraction; 4–8, wash extracts; 9, Ni-NTA column eluant; 10, Amicon filter concentrate. (B) Detection of DSS cross-linked Ac-AChBP complexes.

To generate Ac-AChBP, genomic constructs encoding 6xHis-tagged Ac-AChBP were transformed into bacterial BL21 cells that were then induced for protein synthesis using IPTG. Purification of Ac-AChBP was performed via immobilized metal affinity chromatography (IMAC) on a Ni-NTA column. As shown in Figure 3A, a clean Ac-AChBP protein product was confirmed on an sodium dodecyl-sulfate polyacrylamide gel electrophoresis (SDS-PAGE) gel stained using Coomassie. Ac-AChBP subunits naturally assemble into homopentameric complexes in an aqueous solution.^30^ We assessed the composition of our purified Ac-AChBP within phosphate-buffered saline (PBS), which is also used as the solvent for the protein painting experiment. We observed pentamers of the Ac-AChBP complex at the expected molecular weight (∼140 kDa) following DSS cross-linking (Figure 3B). The pentamer band represented the dominant band on the SDS-PAGE gel relative to the lower monomer band.

Various types of dyes have been shown to bind to native protein complexes, thereby blocking enzymatic digestion of trypsin in liquid chromatography-electrospray ionization (LC/ESI) MS studies.^29^ We used molar access of two covalently binding dye molecules in protein painting of the Ac-AChBP. Atto-425 NHS ester (in DMSO) and 4-nitrobenzenediazonium tetrafluoroborate (in PBS) were used as shown in previous protein painting experiments for the identification of solvent-inaccessible sites within the painted protein complex.^29^

**Figure 4:**
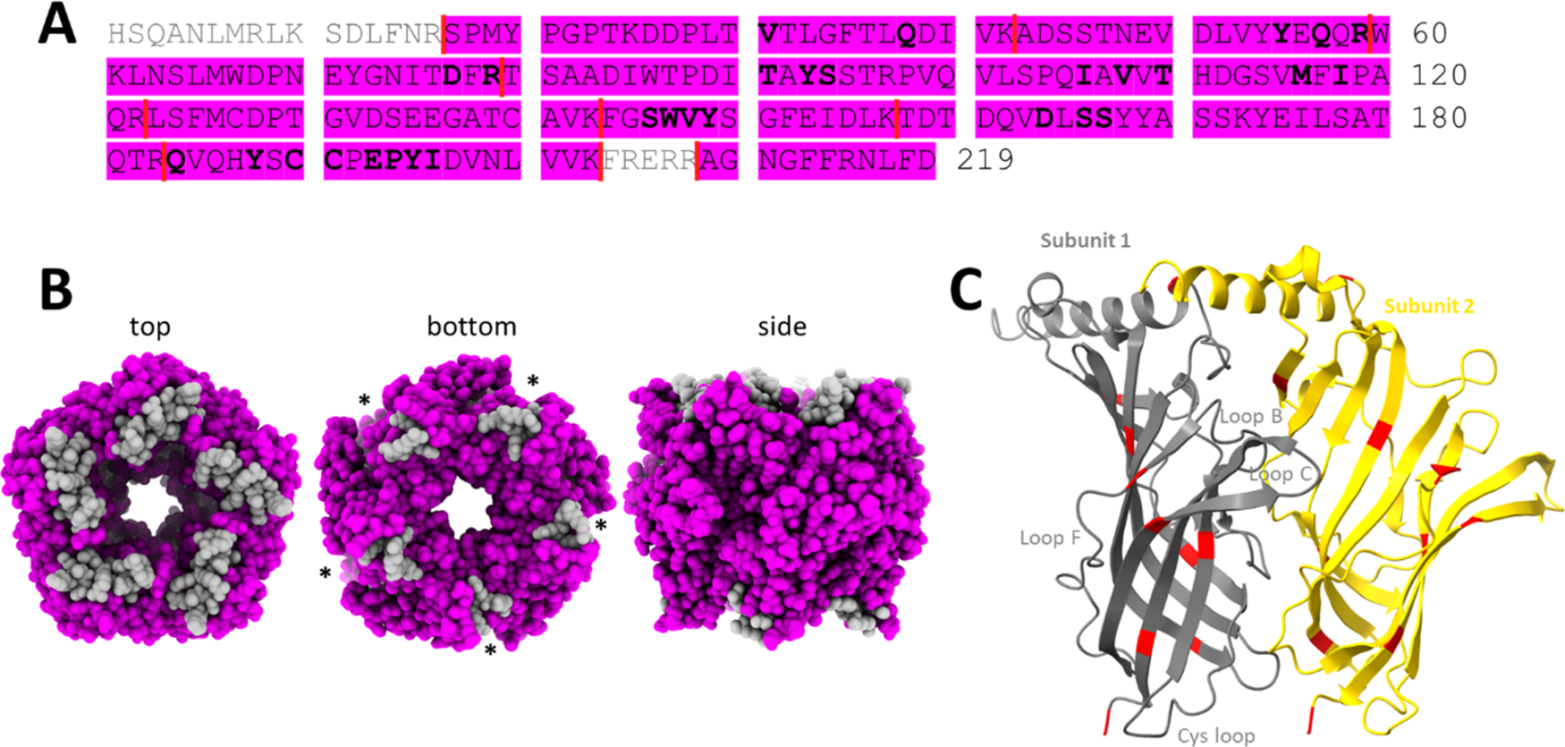
Trypsin coverage of the Ac-AChBP. (A) Sequence coverage of the Ac-AChBP by tryptic EDFs in the protein painting experiment. Red lines mark sites of trypsin cleavage, while bold letters indicate residues involved in ligand binding. (B) Model showing regions of trypsin coverage in the Ac-AChBP. Asterisks indicate subunit interface. (C) Model of the subunit interface showing points of trypsin cleavage (R and K residues).

**Figure 5:**
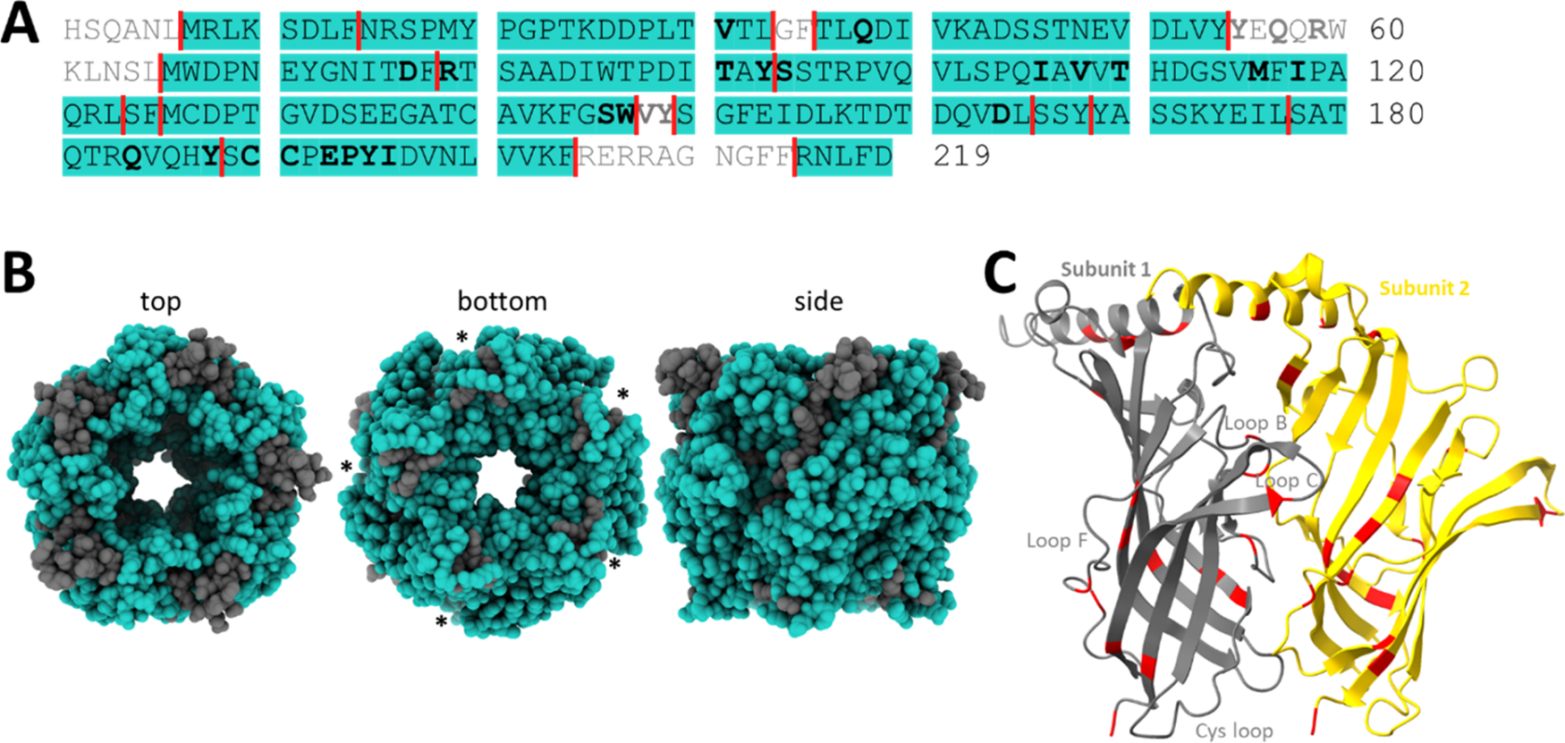
Chymotrypsin coverage of the Ac-AChBP. (A) Coverage of the Ac-AChBP by chymotryptic EDFs in the protein painting experiment. Red lines show sites of chymotrypsin cleavage, while bold letters indicate residues for ligand binding. (B) Structural model of chymotrypsin coverage in the Ac-AChBP. Asterisks indicate subunit interface. (C) Model of the subunit interface showing points of chymotrypsin cleavage (F, Y, W, and L residues).

Since cleavage by different peptidases is expected to yield a varied repertoire of EDFs, we compared sequence coverage of the Ac-AChBP in the MS experiment following trypsin and chymotrypsin digestion. The extent of the Ac-AChBP sequence that was covered following cleavage by trypsin, chymotrypsin, or AspN was also examined in silico using PeptideCutter^31^ (Figure S1A–C). This predictive analysis suggested that all three enzymes allow high coverage (>70%) of the Ac-AChBP protein. Experimental analysis, however, showed that AspN results in only 32% coverage (Figure S1D) and was therefore not used in the study. In MS experiments, digestion with trypsin and chymotrypsin resulted in extensive and complementary coverage of the Ac-AChBP protein (Figures 4 and 5). Specifically, trypsin cleavage (at residues R and K) yielded 90% coverage and encompassed residues involved in ligand binding (Figure 4). Trypsin digestion yielded 13 unique EDFs within the MS analysis (Table 1). In comparison, chymotrypsin cleavage (at F, Y, W, and L) showed 86% coverage of the Ac-AChBP and was found to cut at residues within structural loops (B and C) (Figure 5). Chymotrypsin cleavage was found to generate 25 unique EDFs in the MS analysis (Table 2).

**Table 1:** Trypsin Coverage of the Ac-AChBP^a^

positions in proteins	annotated sequence	modifications	#PSMs
[17–42]	[R].SPMYPGPTKDDPLTVTLGFTLQDIVK.[A]	1 × oxidation [M3]	3
[26–42]	[K].DDPLTVTLGFTLQDIVK.[A]		9
[43–59]	[K].ADSSTNEVDLVYYEQQR.[W]		16
[60–79]	[R].WKLNSLMWDPNEYGNITDFR.[T]	1 × oxidation [M7]	1
[62–79]	[K].LNSLMWDPNEYGNITDFR.[T]	1 × oxidation [M5]	14
[80–122]	[R].TSAADIWTPDITAYSSTRPVQVLSPQIAVVTHDGSVMFIPAQR.[L]	1 × oxidation [M37]	3
[123–143]	[R].LSFMCDPTGVDSEEGATCAVK.[F]	2 × carbamidomethyl [C5; C18]; 1 × oxidation [M4]	3
[144–157]	[K].FGSWVYSGFEIDLK.[T]		6
[158–173]	[K].TDTDQVDLSSYYASSK.[Y]		7
[158–183]	[K].TDTDQVDLSSYYASSKYEILSATQTR.[Q]		7
[174–183]	[K].YEILSATQTR.[Q]		4
[184–203]	[R].QVQHYSCCPEPYIDVNLVVK.[F]	2 × carbamidomethyl [C7; C8]	12
[209–219]	[R].AGNGFFRNLFD.[-]		1

a A List of Tryptic EDFs Identified Using MS Analysis of Ac-AChBP. For each fragment, the number position, peptide sequence, modification, and peptide spectral match (PSM) score are presented.

**Table 2:** Chymotrypsin Coverage of the Ac-AChBP^a^

proteins	annotated sequence	modifications	#PSMs
[7–14]	[L].MRLKSDLF.[N]	1 × oxidation [M1]	7
[15–33]	[F].NRSPMYPGPTKDDPLTVTL.[G]	1 × oxidation [M5]	l2
[36–54]	[F].TLQDIVKADSSTNEVDLVY.[Y]		8
[66–78]	[L].MWDPNEYGNITDF.[R]	1 × oxidation [M1]	15
[73–86]	[Y].GNITDFRTSAADIW.[T]		4
[73–93]	[Y].GNITDFRTSAADIWTPDITAY.[S]		l2
[79–93]	[F].RTSAADIWTPDITAY.[S]		38
[94–102]	[Y].SSTRPVQVL.[S]		l
[94–117]	[Y].SSTRPVQVLSPQIAVVTHDGSVMF.[I]		l
[94–117]	[Y].SSTRPVQVLSPQIAVVTHDGSVMF.[I]	1 × oxidation [M23]	47
[94–123]	[Y].SSTRPVQVLSPQIAVVTHDGSVMFIPAQRL.[S]	1 × oxidation [M23]	l7
[103–117]	[L].SPQIAVVTHDGSVMF.[I]	1 × oxidation [M14]	7
[103–123]	[L].SPQIAVVTHDGSVMFIPAQRL.[S]	1 × oxidation [M14]	10
[118–125]	[F].IPAQRLSF.[M]		6
[124–144]	[L].SFMCDPTGVDSEEGATCAVKF.[G]	2 × carbamidomethyl [C4; C17]; 1 × Oxidation [M3]	3
[126–147]	[F].MCDPTGVDSEEGATCAVKFGSW.[V]	2 × carbamidomethyl [C2; C15]; 1 × oxidation [M1]	2
[150–165]	[Y].SGFEIDLKTDTDQVDL.[S]		4
[153–168]	[F].EIDLKTDTDQVDLSSY.[Y]		2
[169–177]	[Y].YASSKYEIL.[S]		2
[170–177]	[Y].ASSKYEIL.[S]		l
[175–188]	[Y].EILSATQTRQVQHY.[S]		9
[178–188]	[L].SATQTRQVQHY.[S]		6
[189–204]	[Y].SCCPEPYIDVNLVVKF.[R]	2 × carbamidomethyl [C2; C3]	3
[196–204]	[Y].IDVNLVVKF.[R]		l6
[215–219]	[F].RNLFD.[-]		2

a A List of Chymotryptic EDFs Identified Using MS Analysis of Ac-AChBP. For each fragment, the number position, peptide sequence, modification, and PSM score are presented.

### Identification of Protein Binding Sites within Ac-AChBP Using Protein Painting

We tested protein painting MS through a sequential application of the aryl diazonium and NHS-ester protein dyes on Ac-AChBP in the presence or absence of an experimental ligand. We first examined the ability of protein painting to assess interactions between Bgtx and the Ac-AChBP through incubation with the toxin at 10-fold molar access (12 μM) for 1 h at room temperature (RT). Aryl diazonium and NHS-ester protein dyes were then applied sequentially, each for 30 min at RT, and the unbound protein dye was removed via gel filtration. The painted protein complex was denatured, reduced, and alkylated prior to enzymatic digestion by either trypsin or chymotrypsin for the MS analysis. Peptides were compared across sample groups using an abundance ratio for each EDF within the MS spectra. A comparison of the EDF peptide abundance scores between painted and unpainted conditions was performed. Specifically, we measured (1) the ratio of painted Ac-AChBP to unpainted Ac-AchBP as the control condition, in which the abundance ratio should be low as most peptides were painted and have a higher molecular weight than expected based on covalent paint modification, and (2) the ratio of painted Ac-AChBP in the presence of the Bgtx ligand to unpainted Ac-AChBP as the experimental condition. All experiments were performed in triplicate, and statistical analysis was used to identify EDF peptide hits that represent components of the putative binding site identified by the protein painting method (Figure 2).

**Figure 6:**
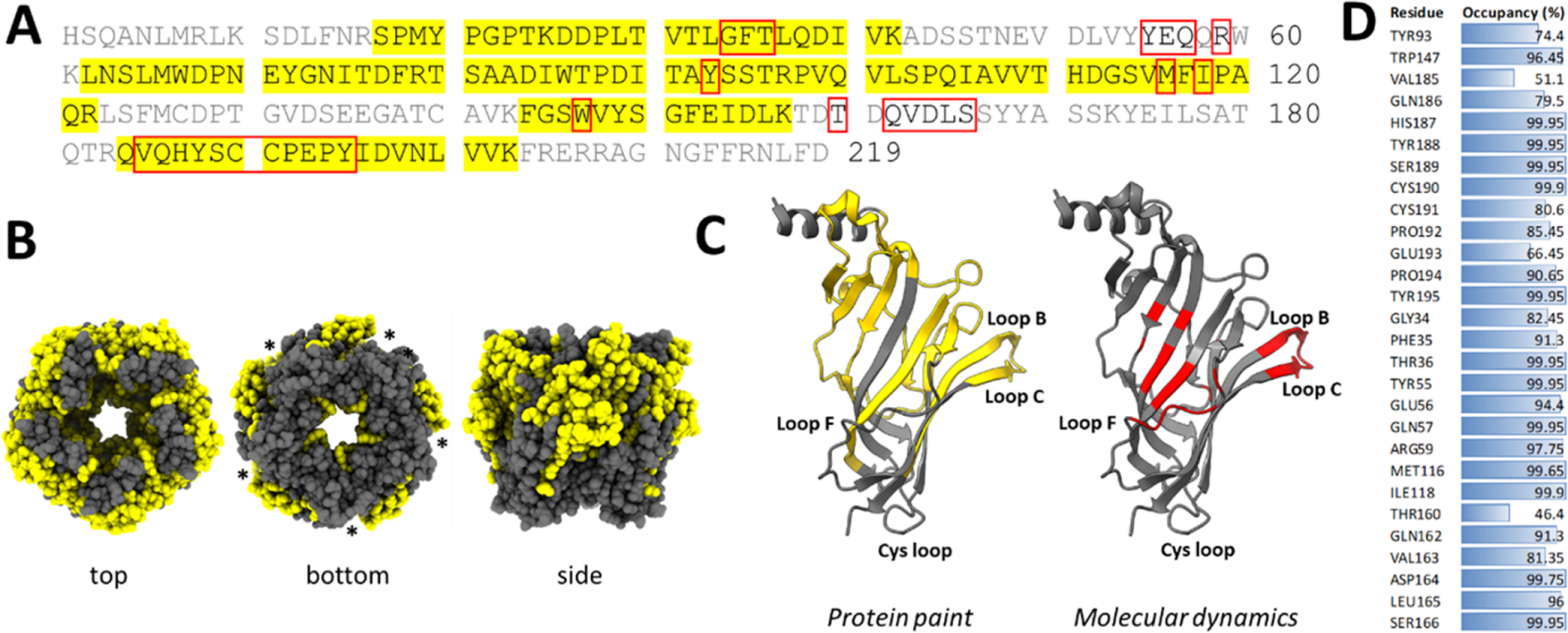
Bgtx binding sites within Ac-AChBP. (A) Identified regions within the Ac-AChBP involved in Bgtx binding based on tryptic and chymotryptic EDF hits (yellow) and high-percentage occupancy (>25%) sites based on molecular dynamics (red boxes). (B) Structural model of Ac-AChBP showing Bgtx binding regions in yellow. Asterisks indicate the interface of adjacent subunits. (C) Structural rendering of the Ac-AChBP subunit showing sites involved in Bgtx binding based on protein painting and molecular dynamics. (D) Percent occupancy of Bgtx binding residues based on molecular dynamic simulations.

As shown in Tables 3 and 4, we detected several potential Bgtx binding regions within Ac-AChBP based on a significant increase in the abundance ratio of 7 unique EDFs. The results are presented for trypsin (Table 3) and chymotrypsin (Table 4) experiments. Peptide fragments with dye inaccessibility in the control group (average abundance ratio >0.25) were excluded. Trypsin digestion resulted in 6 EDF peptide hits, while chymotrypsin digestion yielded only one EDF hit. This chymotrypsin peptide, at sequence positions 103–117, is fully nested within a longer trypsin EDF hit at positions 80–122. Sequence and structural analysis of the identified EDF peptide hits indicates that Bgtx binding involves several extended regions within the Ac-AChBP including the interface of adjacent subunits (Figure 6A,B). A molecular dynamics simulation was conducted to complement the protein painting experiments. Two subunits were simulated to depict a representative interface within the symmetrical homomeric Ac-AChBP complex. As shown in Figure 6C,D (and Table S1), amino acid residues within the Ac-AChBP displaying high binding occupancy to Bgtx through computational analysis were largely within binding sites identified by the protein painting assay. A structural model showing sites of Bgtx binding within the Ac-AChBP based on molecular dynamic results shows an overlap with the findings based on protein painting experiments (Figure 6C).

**Table 3:**
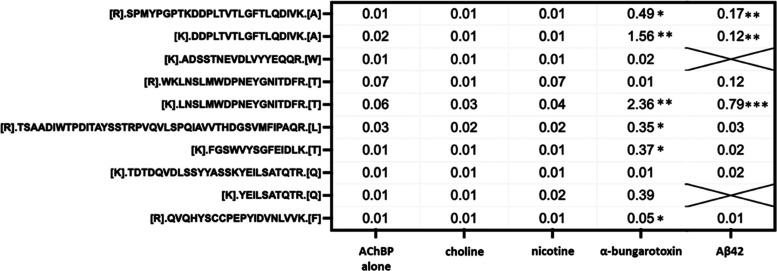
Ratio of Painted to Unpainted Fragments in Ac-AChBP. Geometric Means of Tryptic EDF Ratios^a^

a Geometric Means of Tryptic EDF Ratios. Statistical significance (* *p* < 0.05, ** *p* < 0.01, *** *p* < 0.001) as compared to the ratio of the same peptide in the Ac-AChBP alone condition.

**Table 4:**
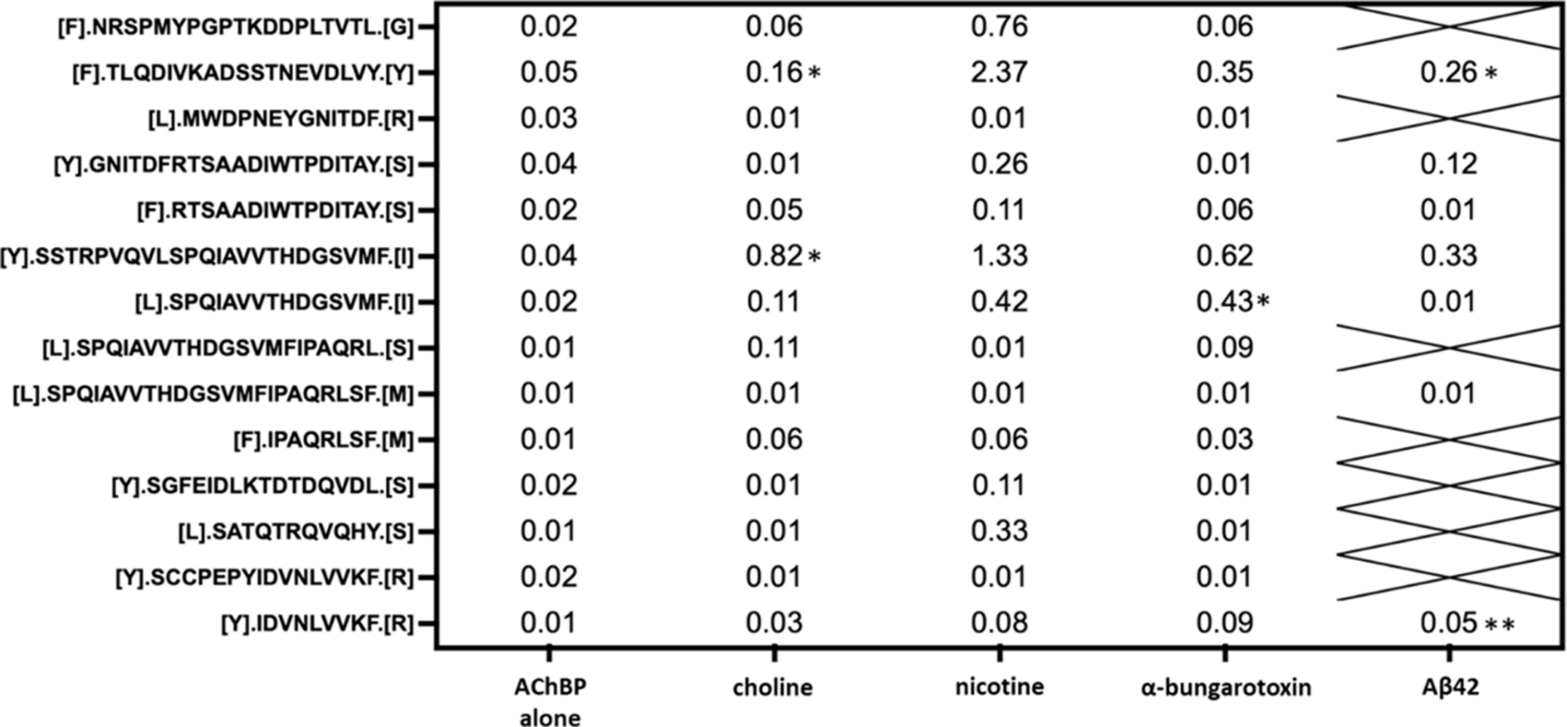
Ratio of Painted to Unpainted Fragments in Ac-AChBP^a^

a Geometric Means of Chymotryptic EDF Ratios. Statistical significance (* *p* < 0.05, ** *p* < 0.01) as compared to the ratio of the same peptide in the Ac-AChBP alone condition.

**Figure 7:**
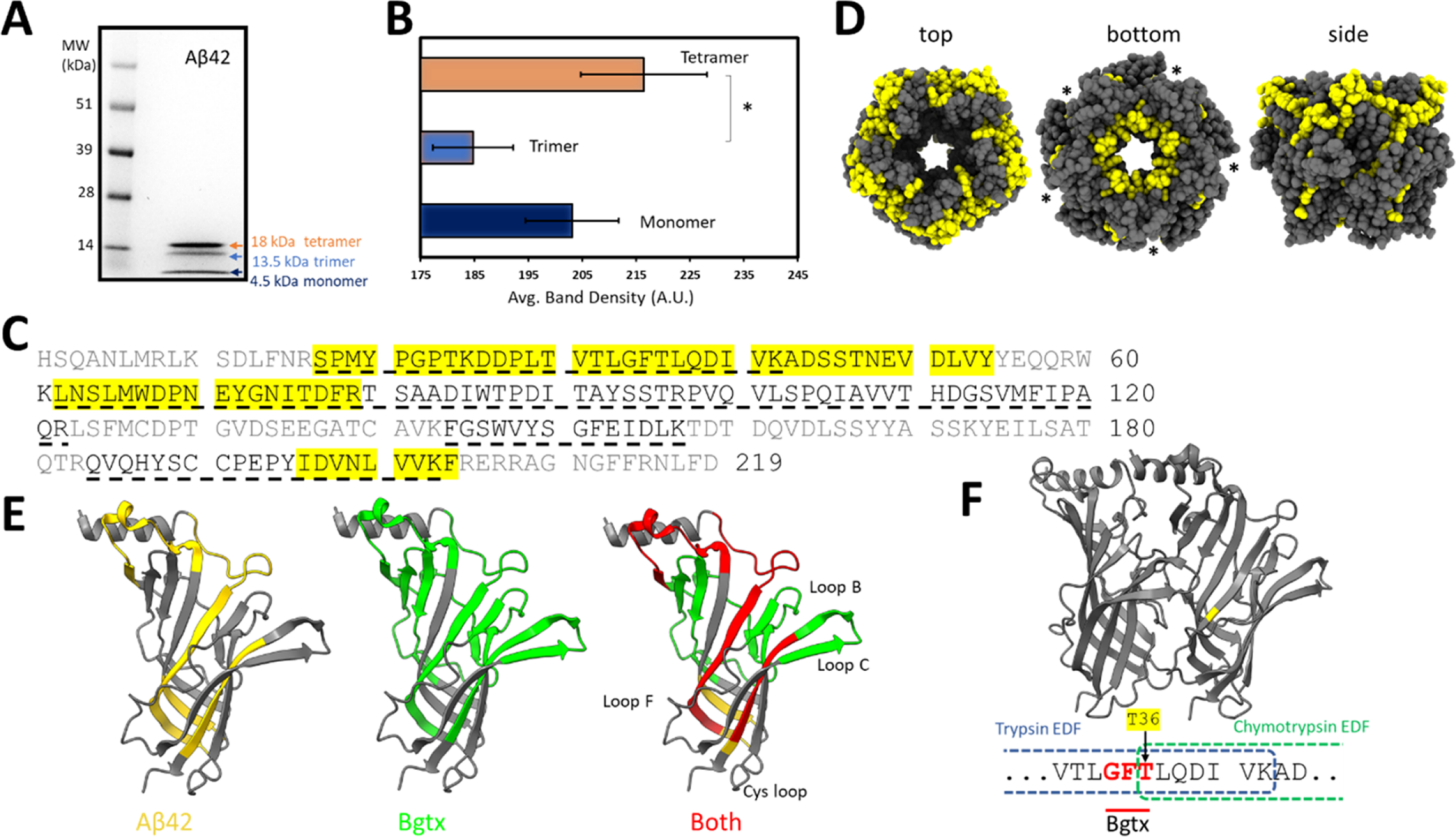
Aβ42 interaction with the Ac-AChBP. (A) Coomassie-stained SDS-PAGE gel showing self-association properties of Aβ42. (B) Average gel band density measures of Aβ42 (*n* = 3, *p < 0.05). (C) Binding regions for Aβ42 within the Ac-AChBP based on tryptic and chymotryptic EDF hits (yellow). Bgtx binding residues are underlined. (D) Structural model of the Ac-AChBP pentamer showing Aβ42 binding regions in yellow. Asterisks indicate the interface of adjacent subunits. (E) Rendering of the Ac-AChBP subunit showing locations of Aβ42 and Bgtx binding. (F) Overlapping site within Aβ42 and Bgtx binding residues.

The amyloid peptide Aβ42 is an intrinsically disordered peptide that can aggregate in higher-order amyloid structures including oligomers and fibrils.^32^ Studies show that Aβ42 can bind α7 nAChR competitively at the cell surface, leading to altered calcium signaling in cultured neuronal cells.^33−35^ We tested the interactions between Aβ42 and Ac-AChBP using protein painting. The solubilization of the Aβ42 peptide was performed as described in a previous method that favors the formation of oligomers with a demonstrated affinity for nAChRs in neural cells.^36^ An SDS PAGE separation was used to assess the aggregation properties of the Aβ42 peptide. As shown in Figure 7A, Aβ42 was found to exist at three distinct molecular weight size bands on the coomassie-stained gel. The bands corresponded to the sizes of Aβ42 monomers (4.5 kDa), trimers (13.5 kDa), and tetramers (18 kDa). Average band density measures across repeated solubilization experiments (*n* = 3) show that tetrameric Aβ42 constitutes the dominant amyloid peptide form within our solution, followed by the monomer (Figure 7B).

We tested for Aβ42 interactions with Ac-AChBP through incubation with the amyloid peptide at a 20-fold molar excess (24 μM), followed by processing and analysis, as described in detail for Bgtx and summarized in Figure 2. As shown in Tables 3 and 4, we detected Aβ42 binding regions within Ac-AChBP based on a significant increase in the abundance ratio of 5 EDF peptide hits. The results are presented for trypsin and chymotrypsin in Tables 3 and 4, respectively. Sequence analysis indicates that Aβ42 interaction sites are relatively localized within the Ac-AChBP. For example, the EDFs associated with Aβ42 binding are found to be shorter in length than those of Bgtx but share some overlapping amino acid residues (Figure 7C). Structurally, Aβ42 binding appears to encompass the interface of adjacent subunits within Ac-AChBP (Figure 7D). A structural rendering of Aβ42 interaction sites within Ac-AChBP highlights protein–protein binding pockets that appear shared with Bgtx (Figure 7E).

### Protein Painting Analysis of Small Molecule Interactions

**Figure 8:**
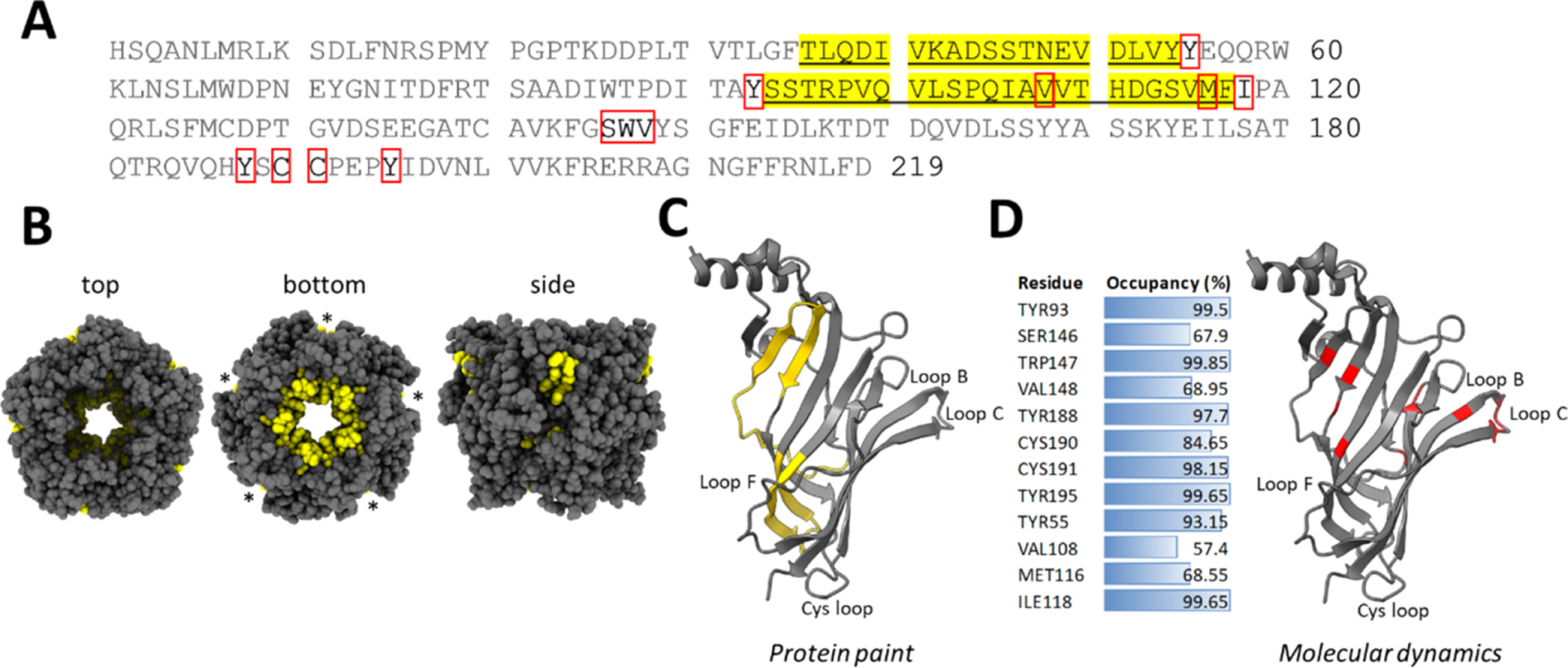
Detection of choline and nicotine binding sites within Ac-AChBP. (A) Binding regions within the Ac-AChBP for choline (yellow) and nicotine (underlined) identified by tryptic and chymotryptic EDF hits. Red boxes indicate residues associated with a high-percentage occupancy score in molecular dynamics simulations. (B) Structural model of the Ac-AChBP pentamer showing the location of choline binding regions in yellow. Asterisks indicate the interface of adjacent subunits. (C) Location of choline-binding within the Ac-AChBP subunit was based on protein painting. (D) Molecular dynamic simulations showing ligand binding residues and their location (in red) within the Ac-AChBP subunit.

Protein painting has been validated for the identification of protein–protein interaction sites within macromolecular complexes.^28,29^ However, protein painting has not been tested for its ability to identify binding sites for small molecule interactions with larger proteins. Conformational changes upon small-molecule ligand binding in nAChRs have been reported,^37^ and thus, we explored the potential for protein painting to identify residues involved in small molecule binding. Soluble Ac-AChBP was incubated with ligands at 10-fold molar (12 μM) excess, followed by processing and MS analysis of EDFs as described earlier (Figure 2). As shown in Tables 3 and 4, we detected choline binding regions within Ac-AChBP based on a significant increase in the abundance ratio of peptide hits within the chymotrypsin experiment. Specifically, the choline presence was associated with a significant increase (*p* < 0.05) in the abundance ratio of two chymotryptic EDFs located at positions 36–54 and 94–117 in the Ac-AChBP sequence. These sequence locations represent sites near the structural interface of two adjacent Ac-AChBP subunits (Figure 8). Protein painting was also conducted to test for interaction sites associated with nicotine presentation. In these experiments, however, the abundance ratio for the identified EDFs did not reach statistical significance due to high variability across experimental repeats (*p* = 0.056). Structural and sequence analyses show that amino acids 36–54 and 94–117 encompass b1 and b5 secondary β sheet structures within the Ac-AChBP complex (Figure 8). Molecular dynamics analysis confirms high occupancy sites for choline and nicotine within the Ac-AChBP complex in these regions (Figure 8C,D and Table S2). Protein painting indicates that small molecules and peptide ligands share some binding sites within the Ac-AChBP. For example, residue positions 36–54 of Ac-AChBP are associated with choline and Aβ42 (Table 4). In addition, the EDF fragment at positions 94–117 that is found to participate in choline-binding contains within it a smaller EDF associated with Bgtx binding (positions 103–117) (Table 4).

## Discussion

The structural similarity, solubility, ligand binding properties, and established use in crystallography and cryo-EM make AChBP a valuable model for studying the structural and ligand binding properties of nAChRs. This paper expands the utility of protein painting MS detection technology in conjunction with structural analysis for the identification of nAChR ligand binding sites using the Ac-AChBP as a model. Ac-AChBP and the freshwater snail *Lymnaea stagnalis* (Ls-AChBP) are among the first AChBP to be crystallized and are well characterized for their structural binding properties. Similar AChBP exist within mollusk and nonmollusk species including *Bulinus truncatus* (Bt-AChBP) and *Capitella teleta* (Ct-AChBP).^38^ Our results suggest that a protein painting strategy is more effective at identifying interactions between the painted protein (i.e., the Ac-AChBP complex) and its peptide ligands (e.g., Bgtx) than small molecule ligands. Results obtained from protein painting studies can guide structure–activity relationship (SAR) analysis in lead compound identification and optimization and may be of value in identifying nearby target sites within the protein complex for further analysis. Protein painting also has the advantage of screening for binding sites when there is no prior knowledge of its properties or location.

### Opportunities in the Development of Protein Painting Mass Spectrometry

The identification of residues involved in ligand binding to Ac-AChBP through protein painting presents an opportunity for the discovery of novel binding motifs and emergent binding structures within the Ac-AChBP. This effort can easily complement structural modeling studies that are often conducted in silico and can serve as a prelude to more expensive and technically demanding atomic resolution techniques such as cryo-EM. Unlike the limitations of full atomic resolution structural analysis, protein painting affords a relatively rapid strategy for detection of solubilized proteins using inexpensive dye reagents and widely available MS instrumentation. However, protein painting detection can also present some immediate limitations in the analysis of the painted complex. For example, enzymatically digested peptides may consist of long stretches of amino acids depending on the sequence of the protein and the location of the peptidase cleavage site. Long EDF may not allow for the identification of precise binding locations within the overall sequence.

An assessment of the sequence coverage at the onset of the experimental design is required for any accurate protein painting analysis. In comparing the digestion profiles of enzymes including trypsin, chymotrypsin, and AspN across the protein painting experiment, we found that both trypsin and chymotrypsin provided high sequence coverage of the Ac-AChBP, thus enabling an ability to identify potential interactions throughout the protein. Despite this initial observation, we found that trypsin digestion is more effective in the discovery of peptide hits for peptide ligands, while chymotrypsin appeared more suitable for identifying interaction sites for small molecules such as choline within the Ac-AChBP. Thus, coverage alone is not sufficient to predict an effective enzyme digestion strategy during the protein painting study, and additional factors such as the potential location of the binding site within generated EDF and the overall structural configuration of the protein interaction site must also be considered. These factors may be guided by pre-existing knowledge based on published works or predictive structural modeling that may guide a hypothesis on the potential for protein interaction sites within the larger complex.

The detection of small molecules capable of functionally binding receptor target sites within proteins is essential to advancing drug design. Our protein painting analysis of a few small molecules (e.g., choline or nicotine) that are known to bind the Ac-AChBP suggests that protein painting is not yet optimized for detecting binding sites with high confidence. This is likely due to several inherent limitations in the adaptation of the protein painting method for small molecule binding site analysis: 1. Limited molecular access of the protein painting dyes to the tight binding grooves where many small molecules bind. This contrasts with the larger, flatter binding sites for most protein–protein interactions. 2. The potential for greater on/off rapid binding kinetics between small molecules and the protein target. 3. The likelihood of limited covalent modification surrounding a binding pocket that is based on very few amino acid residues is often seen for small molecule interaction. These limitations may drive high variability in the EDF results for the small molecule protein painting experiment, as appears to be the case for nicotine in our study, which was associated with a high abundance ratio detection at the same peptide sites as choline (positions 36–54 and 94–117), yet did not reach statistical significance (*p* = 0.056). Although the detection of interaction sites that bind small molecules appears to be somewhat limited for the protein painting approach, future optimization of the chemistry of dye design and MS detection may remedy this.

Protein ligands targeting nAChRs are an important area of research, with evidence that various pathogenic proteins can directly bind to human nAChRs. For example, studies show that α7 nAChRs interact with neurotoxic amyloid proteins such as Aβ42 as well as envelope coat spike proteins for SARS-CoV-2, the gp120 peptide of the human immunodeficiency virus (HIV), and the rabies virus.^16,25,39,40^ Nicotinic receptor targeting through therapeutic peptide design has been explored including various neurotoxins, such as conotoxins, for the treatment of pain and neuroinflammation in animal models.^41,42^ Protein painting provides a promising new platform for the discovery and potential optimization of peptides for drug targeting at the nAChR site. In recent work, protein painting was used to design various new compounds for T-cell regulation.^28,43^

Studies have shown a role for Aβ and especially neurotoxic Aβ42 in AD. However, it is not yet clear how amyloid peptide properties including self-assembly (monomeric vs oligomeric) participate in this disease.^44^ Various methods have been used to address this problem including biocomputational modeling of Aβ structures based on evolutionary sequence alignment algorithms.^45^ Interactions between Aβ42 and nAChRs are well reported in the literature, and functional evidence suggests competitive interactions between Aβ42 and α7 as well as α7β2 nAChR ligands.^46^ Our results show that Aβ42 exhibits some similarity to Bgtx in the protein binding sites within the Ac-AChBP. Specific residues within the Bgtx binding region (Gly34, Phe35, and Thr36) appear to participate in the Aβ42 association (Figure 7F). This evidence is corroborated computationally through structural modeling that demonstrates that Aβ42 and Bgtx bind at residues that are within the interface of two adjacent Ac-AChBP subunits. This result supports existing evidence that Aβ42 acts as a ligand regulator of the nAChR. Sequence alignment of the Ac-AChBP and the human α7 subunit further shows conservation at putative Aβ42 binding residues (positions 56–58 in the α7 nAChR) (Figure 1). It is interesting to consider that the ability of Aβ42 to self-assemble into higher-order structures may result in a disruption to cholinergic neurotransmission through a hindrance of residues at the ligand binding site in the nAChR. In future studies, it will be important to confirm the involvement of these residues in interactions between Aβ42 and Ac-AChBP as well as the α7 nAChR using strategies such as site-directed mutagenesis as well as protein painting.

## Methods

### Expression and Purification of rAc-AChBP

Recombinant Ac-AChBP with the N-terminal (His)6-tag (GenScript) was expressed in *Escherichia coli* BL21(DE3) cells. The Ac-AChBP was incubated in LB media (Miller BP1426–2) containing ampicillin at 37 °C and induced by 1.0 mM isopropyl β-d-thiogalactopyranoside (IPTG) (Millipore Sigma 367–93–1) at OD_600_ of 0.2 at 16 °C as described.^47^ Cells were pelleted and then lysed by sonication for 20 min in lysis buffer containing 20 mM tris, 150 mM NaCl, 10% glycerol, pH 8.0 with DNase, lysozyme, and protease inhibitor tablet (Thermo Fisher A32955). Lysate was centrifuged at 19,000 rpm for 45 min, and then the pellets were resuspended in detergent buffer (20 mM Tris pH 8.0, 150 mM NaCl, 10% glycerol, 10 mM imidazole, 1% Triton-X) with protease inhibitor tablet. The lysate was sonicated for 20 min and then centrifuged at 19,000 rpm for 45 min. The lysate was collected and loaded onto a nickel column. The column was washed with lysis buffer to remove the detergent. The Ac-AChBP was eluted using elution buffer containing 20 mM tris pH 8.0, 150 mM NaCl, 10% glycerol, and 250 mM imidazole. Next, the Ac-AChBP was centrifuged using a 30 kDa Amicon Centrifugal filter for buffer exchange and protein concentration. Lastly, the isolation of Ac-AChBP was determined as >95% pure by SDS-PAGE gel prior to protein painting experiments.

### Chemicals, Peptides, and Cross-Linking

The following ligands were tested in the study: Bgtx (Invitrogen B1601), Aβ42 (Bachem 4014447) solubilized as described in,^36^ Choline (Acros Organics 110290500), and Nicotine (Sigma-Aldrich N3876). Atto-425 NHS ester (Sigma-Aldrich 16805) and 4-nitrobenzenediazonium tetrafluoroborate (TCI N0137) were used as protein dyes as described in Haymond et al., and Luchini et al. Chemical cross-linking was performed using disuccinimidyl suberate (DSS) (Thermofisher A39267) applied at a 1:25 molar ratio (pentamer to cross-linker) for 1.5 h, and the reaction was halted with 1 M tris-HCl. Protein concentrations were determined using the Bradford assay. Proteins were separated on a NuPAGE 4–12% bis-tris gradient gel (Thermo Fisher NP0322BOX), followed by Coomassie staining (MP Biochemicals 04808274).

### Protein Paint and Enzymatic Peptide Cleavage of Protein Complexes

Protein painting has been described in previous studies.^27−29^ Briefly, protein complexes representing the “receptor” Ac-AChBP and the “ligand” were prepared by mixing 2 μg of Ac-AChBP with 10× molar excess of ligand (nicotine, choline, bgtx) or 20× molar excess Aβ42. The receptor and ligand solution were incubated in PBS with gentle rotation for 1 h at room temperature. Protein complexes were dye-pulsed sequentially with 4-nitrobenzenediazonium tetrafluoroborate (5 mg/mL stock in PBS, final concentration 0.3 mg/mL) and then NHS-ester dye (5 mg/mL stock in DMSO, final concentration 0.3 mg/mL) each for 30 min. The unbound dye was removed via gel filtration with Sephadex G25 spin columns (Cytiva 27532501) according to the manufacturer’s directions, and the flow-through was collected and treated with 2 M urea and 10 mM DTT at 37 °C for 15 min. Lysozyme (200 ng) was added as an internal standard to the chymotryptic samples. Protein alkylation was performed with a 15 min incubation with 50 mM iodoacetamide in the dark. The samples were digested overnight at 37 °C with either sequencing-grade trypsin (Promega V5111) or chymotrypsin (Promega V1061) at a protease/protein ratio of 1:10 (w/w). Digestion was halted by adding 5 μL of acetic acid (2% final concentration). The samples were divided into two technical replicates and desalted using C-18 spin columns (G Biosciences 786–931). The purified samples were dried under nitrogen gas prior to liquid chromatography-tandem mass spectrometry (LC-MS/MS) analysis.

### Mass Spectrometry

Mass LC-MS/MS was performed on an Orbitrap Exploris 480 (Thermo Fisher Scientific). Peptide separation was achieved with a reversed-phase PepMap RSLC 75 μm inner diameter x 15 cm long with a 2 μm, C18 resin LC column (Thermo Fisher Scientific) and mobile phases A, a (0.1% aqueous solution of formic acid), and B (0.1% formic acid in an 80% acetonitrile solution). Following sample injection, peptides were eluted through a linear gradient from 5 to 50% B over a duration of 30 min and ramped up to 100% B for an additional 2 min. The flow rate was set at 300 nL/min. The mass spectrometer was employed in a data-dependent mode in which one full MS scan (60,000 resolving power) from 300 to 1500 Da using quadrupole isolation was followed by MS/MS scans in which the top 20 most abundant molecular ions were dynamically selected and fragmented by higher collisional dissociation (HCD) using a normalized collision energy of 27%. “Peptide Monoisotopic Precursor Selection” and “Dynamic Exclusion” (30 s duration) were enabled, as was the charge state dependence, so that only peptide precursors with charge states from +2 to +4 were selected and fragmented by HCD. Tandem mass spectra were searched using Proteome Discover version 2.3 with SEQUEST against the NCBI *E. coli* databases and custom databases containing the recombinant protein sequence. A 1% false discovery rate was used as a cutoff value for reporting peptide-spectrum matches (PSMs) from the database. Protein abundances used in data analysis were determined using label-free protein quantification using the Minora algorithm within Proteome Discoverer.^48^ Trypsin abundance and chymotrypsin abundance normalized to internal standard lysozyme abundance were used for subsequent data analysis. Peptides with an abundance value corresponding to 1 PSM were not included in the analysis. For peptides with painted/unpainted ratios of less than 0.1, 0.1 was imputed, and the geometric mean for each peptide ratio is reported from at least three biological replicates. Significance was determined using the one-tailed *t* test, and the results are displayed in table format created in GraphPad Prism version 10.2.0.

### Computational Studies

AChBP from *A. Californica* was simulated in either a complex with nicotine or Bgtx. The protein in the complex with nicotine PDB ID: 5O87(49) was obtained from the RCSB Protein Data Bank (RCSB PDB).^50^ In the case of the complex with Bgtx, an apo crystal structure of AChBP was used instead of PDB ID: 2BYN(23) and superimposed with Bgtx from a complex with the α7 nAChR PDB ID: 7KOO.^2^ In order to accomplish this, the α carbons of the first chain of α7 were aligned with the α carbons of the first chain of AChBP. Two subunit chains of the protein were simulated, and the first and last five α carbons of each chain were restrained to compensate for the missing subunits. The proteins were parametrized with Amber99SB force field,^51^ solvated in TIP3P water^52^ with 0.15 M NaCl, and the ligand was parametrized with GAFF2 using AmberTools23.^53^ Both systems were simulated for 200 ns using GROMACS 2023.^54^ The root-mean-square deviation (RMSD) for the protein backbone, the bungarotoxin backbone, and the heavy ligand atoms was calculated to check for equilibration. AChBP backbones stayed under 0.3 nm, and the ligands under 0.5 nm over the whole trajectory (Figure S2). Structural figures in the study were generated using ChimeraX software.^55^ Figures were based on PDB structures with the following IDs: 2BYN, 7KOQ, and 2BYQ.^2,23^

## Supporting Information Available

Tables presenting molecular dynamics raw output for Bgtx and nicotine binding sites in the Ac-AChBP; displays PeptideCutter predictions of Ac-AChBP sequence coverage for trypsin, chymotrypsin and AspN, and experimental AspN Ac-AChBP coverage (Figure S1); shows the RMSD for Ac-AChBP backbone with Bgtx or nicotine, the backbone of Bgtx alone, and the heavy atoms of nicotine (Figure S2) (PDF)
