# Supplementary material for: Protein Painting Mass Spectrometry in the Discovery of Interaction Sites within the Acetylcholine Binding Protein
Source: ACS Chem Neurosci. 2024 May 28;15(11):2322–33. doi: 10.1021/acschemneuro.4c00149 (PMC11157483; doi:10.1021/acschemneuro.4c00149)
Supplement: Supplementary file 1 — cn4c00149_si_001.pdf [file cn4c00149_si_001.pdf]

SUPPORTING INFORMATION

Protein painting mass spectrometry in the discovery of interaction sites within the acetylcholine binding protein

Alexandru Graur[1], Amanda Haymond[2], Kyung Hyeon Lee[3], Franco Viscarra[4,5], Paul Russo[2], Alessandra Luchini[2], Mikell Paige[3], Isabel Bermudez-Diaz[4], Nadine Kabbani[1]*

[1]School of Systems Biology, George Mason University, Fairfax VA, 22030
[2]Center for Applied Proteomics and Molecular Medicine, George Mason University, Manassas, Virginia 20110
[3]Department of Chemistry and Biochemistry, George Mason University, Fairfax, Virginia, 20110
[4]Department of Biological and Medical Sciences, Faculty of Health and Life Sciences, Oxford Brookes University, Headington, Oxford OX3 0BP, United Kingdom
[5]Structural Bioinformatics and Computational Biochemistry, Department of Biochemistry, University of Oxford, Oxford OX1 3QU, United Kingdom

*Corresponding author:
4400 University Drive
Fairfax, VA 22030
USA
nkabbani@gmu.edu

Table S1. Raw output of Bgtx binding sites in the Ac-AChBP pentamer using molecular dynamics.

Chain	Average contacts	Residue	Occupancy (%)
A	1	LYS25	0.2
A	2.473790323	TYR93	74.4
A	1.142156863	SER146	10.2
A	5.392431312	TRP147	96.45
A	2.314049587	VAL148	6.05
A	1.134796238	TYR149	15.95
A	1.769230769	SER150	0.65
A	1.2	PHE152	0.25
A	1.837209302	ARG183	2.15
A	2.307692308	GLN184	0.65
A	2.259295499	VAL185	51.1
A	3.183647799	GLN186	79.5
A	8.803901951	HIS187	99.95
A	11.53976988	TYR188	99.95
A	5.906953477	SER189	99.95
A	5.408408408	CYS190	99.9
A	3.32382134	CYS191	80.6
A	5.560561732	PRO192	85.45
A	2.811888638	GLU193	66.45
A	2.444567016	PRO194	90.65
A	7.251625813	TYR195	99.95
A	1	ILE196	0.3
A	1	ASP197	0.05
B	1.793208005	GLY34	82.45
B	3.447973713	PHE35	91.3
B	3.655327664	THR36	99.95
B	4.777388694	TYR55	99.95
B	3.466101695	GLU56	94.4
B	5.92146073	GLN57	99.95
B	4.512531969	ARG59	97.75
B	1	VAL108	0.6
B	2.255895635	MET116	99.65
B	2.62012012	ILE118	99.9
B	1.207207207	ASP159	22.2
B	1.729525862	THR160	46.4
B	1	ASP161	0.1
B	4.286418401	GLN162	91.3
B	1.711739398	VAL163	81.35
B	5.357894737	ASP164	99.75
B	6.066666667	LEU165	96
B	3.895447724	SER166	99.95
B	1.396946565	SER167	19.65
B	1	TYR168	0.05
B	1.090909091	ILE176	0.55
B	1.22295082	LEU177	15.25
B	1.519480519	SER178	7.7
B	1.111111111	ALA179	0.45

Table S2. Raw output of nicotine binding sites in the Ac-AChBP pentamer using molecular dynamics.

Chain	Average contacts	Residue	Occupancy (%)
A	3.38040201	TYR93	99.5
A	1.124447717	SER146	67.9
A	10.34702053	TRP147	99.85
A	2.50688905	VAL148	68.95
A	1	TYR149	0.15
A	1.227748691	GLN186	19.1
A	6.452917093	TYR188	97.7
A	1.09509746	CYS190	84.65
A	1.50585838	CYS191	98.15
A	1.5	PRO192	0.1
A	1.479020979	GLU193	14.3
A	7.366783743	TYR195	99.65
B	1.666666667	GLN38	0.15
B	4.082662373	TYR55	93.15
B	1.894117647	GLN57	4.25
B	1	ARG79	0.3
B	1	ILE106	0.35
B	1.114982578	VAL108	57.4
B	1.298322392	MET116	68.55
B	1.666666667	PHE117	0.15
B	3.68489714	ILE118	99.65
B	1	SER167	0.25

A **Predicted Trypsin Coverage (*in silico* PeptideCutter: 74%)**

```
HSQANLMRLK SDLFNRSPMY PGPTKDDPLT VTLGFTLQDI VKADSSTNEV DLVYYEQQRW 60
KLNSLMWDPN EYGNITDFRT SAADIWTPDI TAYSSTRPVQ VLSPQIAVVT HDGSVMFIPA 120
QRLSFMCDPT GVDSEEGATC AVKFGSWVYS GFEIDLKTDT DQVDLSSYYA SSKYEILSAT 180
QTRQVQHYSC CPEPYIDVNL VVKFRERRAG NGFFRNLFD 219
```

B **Predicted Chymotrypsin Coverage (*in silico* PeptideCutter: 93%)**

```
HSQANLMRLK SDLFNRSPMY PGPTKDDPLT VTLGFTLQDI VKADSSTNEV DLVYYEQQRW 60
KLNSLMWDPN EYGNITDFRT SAADIWTPDI TAYSSTRPVQ VLSPQIAVVT HDGSVMFIPA 120
QRLSFMCDPT GVDSEEGATC AVKFGSWVYS GFEIDLKTDT DQVDLSSYYA SSKYEILSAT 180
QTRQVQHYSC CPEPYIDVNL VVKFRERRAG NGFFRNLFD 219
```

C **Predicted AspN Coverage (*in silico* PeptideCutter: 81%)**

```
HSQANLMRLK SDLFNRSPMY PGPTKDDPLT VTLGFTLQDI VKADSSTNEV DLVYYEQQRW 60
KLNSLMWDPN EYGNITDFRT SAADIWTPDI TAYSSTRPVQ VLSPQIAVVT HDGSVMFIPA 120
QRLSFMCDPT GVDSEEGATC AVKFGSWVYS GFEIDLKTDT DQVDLSSYYA SSKYEILSAT 180
QTRQVQHYSC CPEPYIDVNL VVKFRERRAG NGFFRNLFD 219
```

D **AspN: 32% experimental sequence coverage**

```
HSQANLMRLK SDLFNRSPMY PGPTKDDPLT VTLGFTLQDI VKADSSTNEV DLVYYEQQRW 60
KLNSLMWDPN EYGNITDFRT SAADIWTPDI TAYSSTRPVQ VLSPQIAVVT HDGSVMFIPA 120
QRLSFMCDPT GVDSEEGATC AVKFGSWVYS GFEIDLKTDT DQVDLSSYYA SSKYEILSAT 180
QTRQVQHYSC CPEPYIDVNL VVKFRERRAG NGFFRNLFD 219
```

Figure S1. A-C) Ac-AChBP coverage *in silico* by PeptideCutter for trypsin, chymotrypsin, and AspN. D) Experimental coverage of the Ac-AChBP by AspN EDFs.

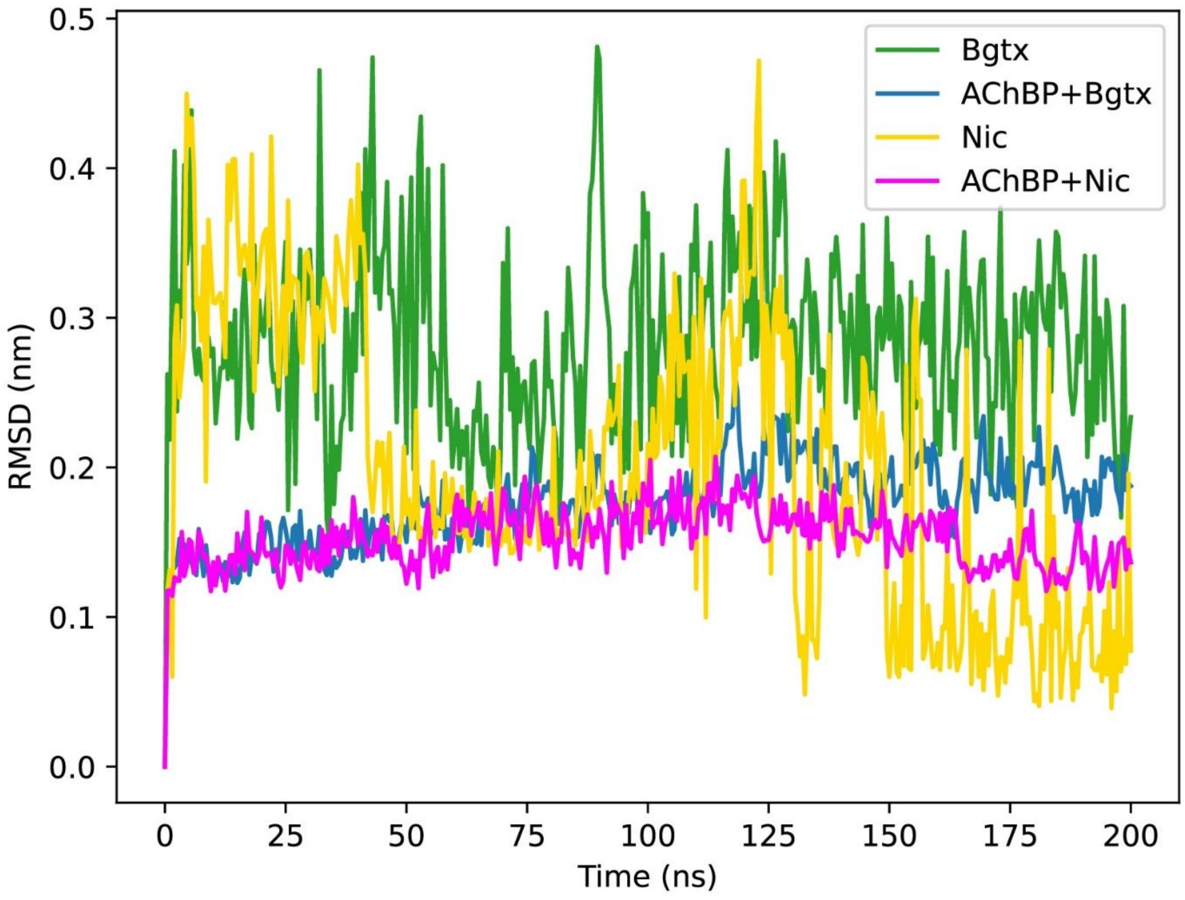

Figure S2. Root-mean-square deviation (RMSD) for the backbone of Ac-AChBP in combination with Bgtx or nicotine, the backbone of Bgtx, and the heavy atoms of nicotine along the trajectory time.
